# Relationship of 24-Hour Movement Behaviors with Weight Status and Body Composition in Chinese Primary School Children: A Cross-Sectional Study

**DOI:** 10.3390/ijerph19148586

**Published:** 2022-07-14

**Authors:** Lin Zhou, Wei Liang, Yuxiu He, Yanping Duan, Ryan E. Rhodes, Hao Liu, Hongmei Liang, Xiaowei Shi, Jun Zhang, Yingzhe Cheng

**Affiliations:** 1 School of Physical Education, Hebei Normal University, Shijiazhuang 050024, China; zlin5198@126.com (L.Z.); richard_h0439@163.com (H.L.); lianghongmei5627@163.com (H.L.); shixiaowei0001@hotmail.com (X.S.); zj572767111@163.com (J.Z.); 2 Key Laboratory of Measurement and Evaluation in Exercise Bioinformation of Hebei Province, Shijiazhuang 050024, China; chyzh77@126.com; 3 Center for Health and Exercise Science Research, Hong Kong Baptist University, Hong Kong 999077, China; wliang1020@hkbu.edu.hk (W.L.); duanyp@hkbu.edu.hk (Y.D.); 4 Department of Sport, Physical Education and Health, Hong Kong Baptist University, Hong Kong 999077, China; 5 School of Exercise Science, Physical and Health Education, University of Victoria, Victoria, BC V8W 2Y2, Canada; rhodes@uvic.ca; 6 Zhihui Primary School, Shijiazhuang 050024, China

**Keywords:** physical activity, sedentary time, sleep, obesity, body fat percent, fat-free mass, skeletal muscle mass, children

## Abstract

24 h movement behaviors, specifically physical activity (PA), sedentary behavior, and sleep, play a crucial role in the prevention and intervention of childhood obesity. This study aimed to examine the association of 24 h movement behaviors with weight status and body composition among Chinese primary school children. Using a random stratified sampling, 978 eligible participants (9.1 ± 1.4 years, 53.2% boys) were recruited from 1 May to 15 July 2021. Demographics included children’s age, gender, grade, parents’ education level, and household income. Movement behaviors were measured by validated self-reported scales. Weight status and body composition (percent of body fat, PBF; fat-free mass, FFM; skeletal muscle mass, SMM) were measured objectively. Results indicated that participants who were younger, boys, and at lower grade showed higher guidelines adherence. PA was inversely associated with PBF, while screen time (ST) was positively associated with overweight/obesity risk and FFM. Sleep showed no association with any health indicators. Meeting the behavioral guidelines was associated with better weight status and lower PBF, yet not with FFM and SMM. Interventions to improve the Children’s weight status and PBF should involve enhancing their overall movement behaviors and considering their demographic differences. More research on examining the association of movement guidelines adherence with body composition indicators is needed.

## 1. Introduction

Childhood obesity is a severe public health problem worldwide [1]. Despite increasing concern and awareness, the prevalence of childhood obesity remained high [2]. Globally, over 340 million children and adolescents were estimated to be overweight and obese in 2016, with an approximately tenfold increase since 1975 [3,4]. In China, the rate of overweight and obesity in children aged 6–17 years was around 19% in 2020, with an increase of 0.5% every year since 2015 [5]. Childhood obesity is highly likely to continue into adulthood [6], increasing the risk of various physical and psychological diseases (e.g., cardiovascular diseases, type-2 diabetes, and mental disorders) and posing massive medical and socioeconomic burdens to society [7,8]. Therefore, the prevention of childhood obesity is imperative to public health and economic sustainability.

Previous evidence demonstrated that regular physical activity (PA) (especially with moderate-to-vigorous intensity), limited screen time (ST) and proper sleep duration with good quality, collectively referred to as 24 h movement behaviors, are crucial modifiable correlates of weight status (i.e., overweight/obese or not, as identified by body mass index, BMI) and body composition (e.g., PBF, FFM, SMM) in children and adolescents [2,9,10]. For instance, engaging in a larger amount of PA (especially with high intensity) was found be significantly associated with a lower risk of overweight/obesity, lower levels of PBF and higher levels of FFM and SMM in children from different countries [11,12]. ST was also evident to be positively associated with the weight status and PBF and negatively correlated with the FFM and SMM in children [13,14,15,16]. For sleep duration, it showed an inverse association with risks of overweight/obesity and percentage of body fat [16,17], while limited studies have examined the relationship between sleep duration and FFM and SMM in children [18].

Alongside the individual impact of PA, ST and sleep, the combined effect of these movement behaviors in a 24 h day on children’s weight status and body composition indicators has raised increasing concerns [19,20]. Most noteworthy, Canadian 24 h movement guidelines were formulated based on the theory of time-use epidemiology [21,22], proposing specific integrated recommendations for moderate-to-vigorous physical activity (MVPA), ST and sleep in children and adolescents [23]. These guidelines recommend that children and adolescents (5–17 years) should do at least 60 min of MVPA/day, limit the screen time (ST) to no more than 2 h/day and sleep 9–11 h (6–13 years)/8–10 h (14–17 years) every day [24]. An increasing number of studies have been conducted to examine the association between adherence to the Canadian 24 h movement guidelines and health among children and adolescents (e.g., from America, Canada, Japan, Spain and China) [20,24]. A recent systematic review summarized evidence from 20 studies and found that adhering to the 24 h movement guidelines was beneficial for a wide variety of health indicators (especially for weight status) in children [20].

Despite the important evidence found in Wang (2022) [20], the review also highlighted current limitations of prior work. Most existing studies were conducted in Western countries/regions while research in Chinese children and adolescents, especially in Chinese primaries, is limited. While there is some evidence suggesting that Chinese adolescents who meet 24 h movement guidelines have lower obesity and overweight [2,10], there is a fragmented understanding of the association between the guideline adherence and weight status in primary school children covering all grades.

Although weight status (BMI) is an acceptable measure for overall adiposity of children, it does not capture information about body composition, such as PBF, FFM and SMM [25], which can be reliably quantified by techniques, such as dual-X ray absorptiometry, computerized tomography and bioelectrical impedance analysis [26]. For children and adolescents who are at a rapid period of physical growth, accurate measurement of body composition and identification of its influential factors (e.g., behavioral correlates) are important and conducive to timely and comprehensive understanding of nutritional status, hypertension metabolic disease risks and overall adiposity [26,27,28]. Nevertheless, there is scarce evidence examining the association between 24 h movement guideline compliance and body composition parameters (e.g., PBF, FFM and SMM) in children, especially in China [19,20,29].

In addition, understanding the factors affecting the compliance of 24 h movement behaviors is crucial for developing effective interventions and policymaking. A series of demographic variables were emphasized in previous theories (e.g., socioecological model) and demonstrated to be associated with children’s 24 h movement and diverse health outcomes [2,20,30]. For example, previous evidence demonstrated that gender, age, grade, parental education level and household income were salient correlates [31,32,33]. However, most of the existing studies that examined the contribution of these demographics on children’s behaviors and health were conducted in Western countries [31,32,33], while evidence of Chinese primary school children covering grade 1–6 is still limited. We, therefore, included these five demographic variables in the examination in our current study.

Given the above, this study aimed to (1) investigate the demographic characteristics of adhering to the 24 h movement guidelines among Chinese primary school children (grade 1–6); (2) examine the association of individual movement behaviors with weight status and body composition; and (3) examine the association of adhering to the 24 h movement guidelines with weight status and body composition indicators. We hypothesized that (1) adherence to the 24 h movement guidelines would differ significantly in demographics (i.e., children’s age, gender, grade, parents’ education level and household income); (2) higher PA, less ST and guideline-level sleep duration would be significantly associated with lower risks of overweight and obesity, lower levels of PBF, higher levels of FFM and higher levels of SMM; and (3) adhering to the 24 h movement guidelines would be significantly associated with low risks of overweight and obesity, lower levels of PBF, higher levels of FFM and higher levels of SMM.

## 2. Materials and Methods

### 2.1. Study Design and Participants

This cross-sectional study was embedded in the baseline survey of a large research project funded by the National Social Science Fund of China (National Office for Philosophy and Social Sciences, Beijing, China; Ref. No.: 19200526). Using a random stratified sampling approach, four public primary schools (grade 1–6) were recruited from four major districts in Shijiazhuang city (one from each district) in Hebei, China. For each school, two classes of students were randomly selected from each grade. Eligible participants met the inclusion criteria, including: (1) no restriction of physical mobility (e.g., physical disabilities); (2) no cognitive or mental disorders; and (3) no intellectual disability or testing risk of body composition.

### 2.2. Procedure and Quality Control

This study was conducted according to the Declaration of the Helsinki World Medical Association [34]. The study protocol was approved by the Institutional Review Board of Hebei Normal University, Shijiazhuang, China (ref. No. 2021LLSC051) and permission to conduct the study was obtained from the teachers and principals of participating schools. All participants participated in the study voluntarily. Written informed consent form was completed by both children and parents prior to the study commencement.

The data collection was conducted by two experienced researchers with assistance of the head teacher of participating class between 1 May and 15 July 2021 (after lockdown). Considering the limited cognitive ability of children in grade 1–3, the head teacher invited the students’ parents to complete the questionnaires at home [35]. For children in grade 4–6, they were asked to complete the questionnaires themselves before or after the class in the classroom setting. The duration of each questionnaire survey was around 15 min. In addition, the height, weight and body composition were measured by class between 8.00 a.m. and 9.00 a.m. at a multi-function sport room (5–10 min/person).

### 2.3. Measures

#### 2.3.1. Demographic Information

Demographic information included children’s age, gender, grade (primaries 1–6), ethnicity (Han or others), parental educational level (below college; college or above) and yearly household income (low: RMB <84,000; medium: RMB 84,000–132,000; high: RMB >132,000) [36].

#### 2.3.2. 24 h Movement Behaviors

PA, ST and sleep were measured using reliable and valid items derived from the Health Behavior in School-aged Children survey questionnaire which had been widely applied in previous studies with Chinese primary children (Cronbach’s alpha = 0.66–0.83) [2,37,38,39]. For PA, participants were asked to report “how many days they engaged in light physical activity at least 60 min on weekdays during the past week” and “how many days they engaged in moderate and vigorous physical activity (MVPA) at least 60 min on weekdays during the past week”. To help participants understand the difference between light PA and MVPA, instructive examples and explanations were provided (e.g., MVPA refers to “any kind of physical activities increasing your heart rate and breathing frequencies during a period, such as brisk walking, hiking, riding a bike and dancing). Responses were given on a 7-point Likert scale, from 0 = none to 7 = 7 days. The total PA (MET-hour/day) was defined as the sum of light PA and MVPA. For ST, participants were asked to report the hours they spent on three scenarios of being sedentary in their leisure time on weekdays and weekends during the past week, including (1) watching TV or movies, (2) playing video games and (3) activities using electronic screen-based devices. Responses were given on a 5-point Likert scale for each scenario (none, about 0.5, 1, 2 or 3 h or more). For sleep, participants were asked to report their usual nightly sleep duration (in hours) on a normal day.

According to the Canadian 24-hour Movement Guidelines [23], participants were categorized as adherence or non-adherence to the guidelines. Particularly, meeting the PA guideline requires that participants report 7 days with at least 60 min of MVPA daily, meeting the ST guideline requires daily ST ≤ 2 h per day and for sleep guideline, 9–11 h/day are recommended for 6–13-year group and 8–10 h/day are recommended for 14–17-year group.

#### 2.3.3. Weight Status and Body Composition

Following a standardized protocol [40], participant’s body weight and body height were measured using calibrated medical digital scales (RGT-140, Changzhou, China) and portable stadiometer (GMCS-I, Beijing, China) to the closest 0.05 kg and 0.1 cm, respectively. BMI was calculated as body mass (kg) divided by body height squared (m^2^). The Chinese sex-specific and age-specific BMI cutoffs points were used to define overweight and obese participants [41].

A bio-impedance analysis (BIA) was conducted using Portable body composition analyzer (InBody230, Seoul, South Korea) and Lokin Body 120 software (DMS-BIA technology; InBody Co., Seoul, South Korea) to estimate body composition, including percent of body fat (PBF), fat-free mass (FFM, kg) and skeletal muscle mass (SMM, kg). The instrument was validated against dual-energy X-ray absorptiometry for school-age children with satisfactory results for estimating body fat [42]. Participants were measured wearing underwear without shoes, and guidelines on how to prepare a child for measurement were given in the paper form to parents and teachers (e.g., the test was conducted between 8.00 a.m. and 9.00 a.m. on an empty stomach. Participants should avoid severe physical activity one day prior to the measurement and they should be adequately hydrated) [43]. Body composition data were collected at the multi-function sport room of the participating schools by two qualified staff, with the air-conditioning temperature controlled at 20 °C and humidity controlled at 50%.

### 2.4. Statistical Analyses

Data analyses were conducted using IBM SPSS 27.0 (Armonk, NY, USA). Invalid and abnormal values (above ±3 standard deviation of mean values) of all independent and dependent variables were cleaned prior to the analysis [44]. Descriptive statistics including mean, standard deviation (SD) and percentage (%), were used to present the characteristics of the study sample. Demographic patterns of adhering to 24 h movement guidelines were examined using independent *t*-test or Chi-square test. Generalized linear mixed models were used to examine the associations of individual variables (demographics and individual movement behavior) with weight status (overweight/obese or not) and body composition (PBF, FFM and SMM) as well as to examine the associations of meeting the 24 h movement guidelines with weight status and body composition, controlling for all demographic covariates. Based on a retrospective power calculation, with an alpha of 0.05 and a statistical power of 80%, the final sample size was adequate to detect an effect size *f*^2^ of 0.01 in the regression model [45]. The statistical significance level was set at *p* < 0.05 (two-tailed). Cohen’s *d* was calculated as a measure of effect size for difference tests, with 0.2, 0.5 and 0.8 indicating a small, medium and large effect size, respectively [46,47].

## 3. Results

### 3.1. Sample Characteristics

**Figure 1:**
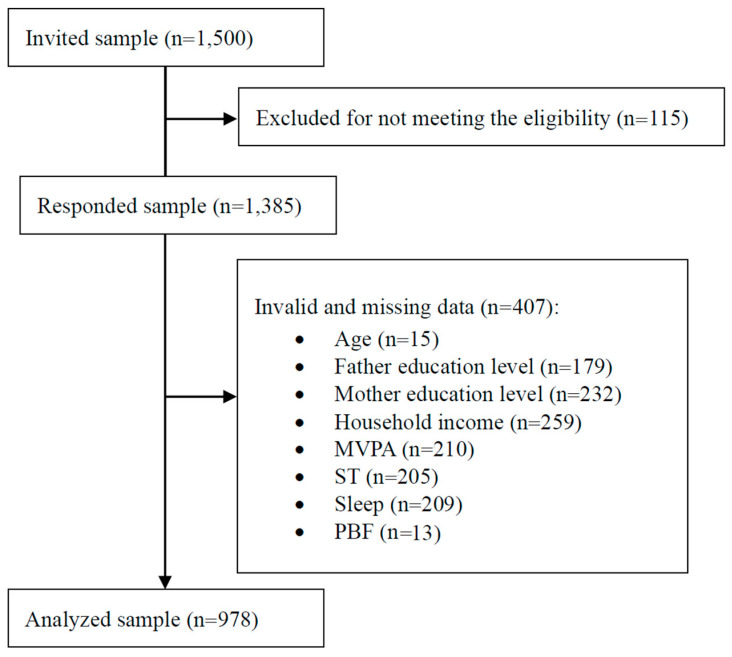
STROBE diagram of study process; MVPA = moderate and vigorous physical activity; ST = screen time; PBF = percent of body fat.

We contacted 1500 participants and received a 92.3% response rate. After cleaning invalid and abnormal values of all independent and dependent variables (Figure 1), in total, 978 eligible participants (53.2% boys; 9.11 ± 1.39 years) were included in the final analysis.

Table 1 shows the descriptive characteristics of the study sample. Overall, 29.3% of participants were overweight or obese. Mean values of PBF, FFM and SMM were 21.01 ± 9.53%, 26.85 ± 6.57 kg and 13.77 ± 3.86 kg, respectively. Participants who were younger, boys, at lower grade and not overweight/obese, lower levels of PBF, FFM and SMM showed higher adherence to the 24 h movement guidelines (all *p* < 0.05).

**Figure 2:**
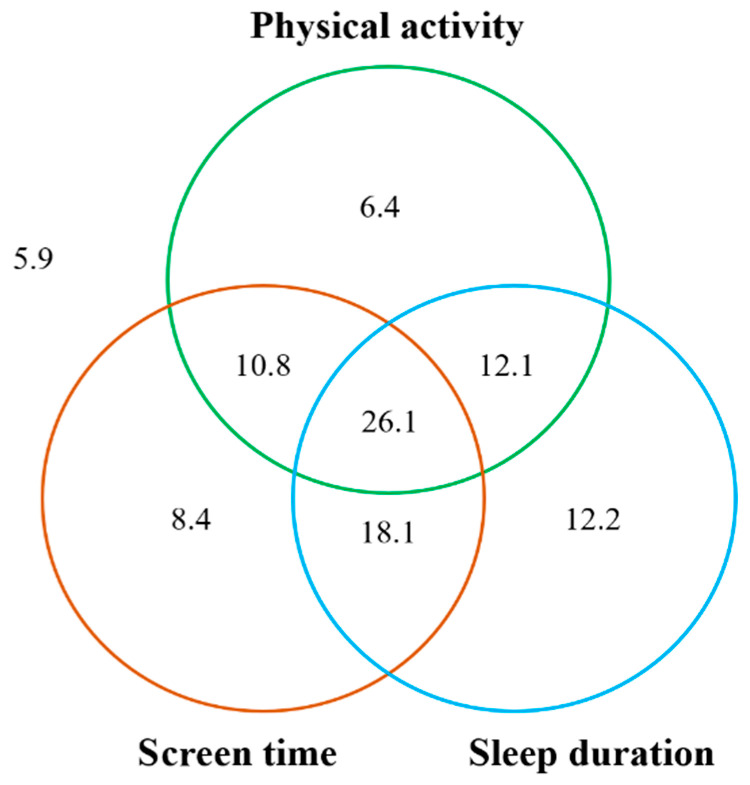
The numbers within each circle are added to the proportion of children meeting each individual guideline (i.e., 55.4% for physical activity, 63.4% for screen time and 68.4% for sleep duration). The total non-overlap area of each circle represents the proportion of children meeting one of the three guidelines (i.e., 6.4% + 8.4% + 12.2% = 27.0%). The total overlap areas of two circles represents the proportion of children meeting two guidelines of the three guidelines (i.e., 10.8% + 18.1% + 12.1% = 41.0%). The overlap area of three circles represents the proportion of children meeting all three guidelines (i.e., 26.1%). The outside area of the circle represents the proportion of children not meeting any of the guidelines (i.e., 5.9%).

The percentage of participants adhering to individual movement guidelines was 55.4% for MVPA, 63.4% for ST and 68.4% for sleep, respectively. Percentages of participants adhering to a combination of two movement behaviors ranged from 36.9 to 44.2%, while 26.1% of participants met all three behavioral recommendations (Figure 2).

### 3.2. Associations of Individual Movement Behavior with Weight Status and Body Composition

The associations of individual movement behavior with weight status and body composition are presented in Table 2 and Table 3. Children who were overweight or obese showed higher levels of PBF, FFM and SMM compared with those who had normal weight status. After adjusting for demographics and/or weight status, engaging in more daily PA of all intensities was significantly associated with lower levels of PBF (*B* = −0.03, *p* < 0.001), while a greater amount of ST was significantly associated with a higher likelihood of overweight/obesity (*OR* = 1.22, *p* = 0.013) and with a higher level of FFM (*B* = 0.27, *p* = 0.043) among study samples. Sleep duration was not significantly associated with any health indicators (all *p* > 0.10).

### 3.3. Associations of Movement Guidelines Adherence with Weight Status and Body Composition

Table 4 shows the associations of meeting the 24 h movement guidelines (individual and in combination) with weight status and body composition. Adhering to the PA guideline was significantly associated with a lower risk of overweight/obesity (*OR* = 0.66, *p* = 0.004) and lower levels of PBF (*B* = 0.27, *p* < 0.001). Adhering to the ST guideline was significantly associated with a lower risk of overweight/obesity (*OR* = 0.72, *p* = 0.031), while meeting the sleep guideline was not associated with these four health indicators (all *p* < 0.10). Meeting PA + ST and PA + sleep guidelines was associated with a lower likelihood of overweight/obesity (*OR*_PA + ST_ = 0.67, *p* = 0.008; *OR*_PA + Sleep_ = 0.67, *p* = 0.011) and a lower level of PBF (*B*_PA + ST_ = −1.33, *p* = 0.001; *B*_PA + Sleep_ = −1.14, *p* = 0.004). Participants who met all three movement guidelines were less likely to be overweight/obese compared to those who did not (*OR* = 0.63, *p* = 0.008). Non-significant correlations were found between meeting the combination of movement guidelines and FFM and SMM (all *p* ≥ 0.05). The dose–response relationships were only identified between the number of guidelines adhered to and weight status (*OR* = 0.78, *p* = 0.01) and PBF (*B* = −0.85, *p* = 0.001).

## 4. Discussion

This study investigated the demographic characteristics in 24 h movement guideline adherence and examined the associations of individual movement behavior (PA, ST and sleep), guideline adherence with weight status (overweight/obesity or not) and body composition indicators (PBF, FFM and SMM) among Chinese primary school children. Results indicated that primary school children who were younger, boys and at lower grade showed a higher compliance with the 24 h movement guidelines (Hypothesis 1). For the association of individual movement behavior with weight status and body composition indicators, engaging in more total PA was associated with less PBF, while having more ST was associated with higher risks of overweight/obesity and higher levels of FFM. Sleep duration showed no significant correlation with any health outcome indicators in the multivariate analyses (Hypothesis 2). In addition, primary school children who met more Canadian 24 h movement guidelines showed a lower risk of overweight and obesity and lower levels of PBF, while the number of guidelines adhered to was not significantly associated with FFM and SMM in the study samples (Hypothesis 3).

While comparing this study with previous studies, the time frame of study implementation should be considered. The data collection was conducted during the COVID-19 pandemic, where several preventive measures were still being implemented by local government and primary schools in Shijiazhuang city (e.g., emergent school closures, physical distancing, compulsory quarantine, etc.). For the guideline adherence, we found slightly higher percentages of meeting the individual behavioral guideline and all movement guidelines compared with the children sample of a previous national survey in China [2]. The discrepancy might be attributed to several factors, including the time of data collection (May to July 2021 vs. October to December 2017) and different age groups of participants (primaries 1–6 vs. primaries 4–6). Interestingly, our findings and previous evidence with Chinese children and adolescents consistently showed a higher prevalence of meeting the 24 h movement guidelines compared to samples from Western countries (e.g., Canada) [20,24,48]. This might be due to the impacts of school environment and governmental policy in China (e.g., mandatory physical active breaks in schools, national physical fitness promotion campaigns) [49]. However, we found there is still a large proportion of Chinese primary school children who reported unhealthy movement behaviors, highlighting the long-term need of effective behavioral interventions and policymaking.

Regarding Hypothesis 1 (i.e., guideline adherence would differ significantly in several demographics), our findings only supported significant differences in age, gender and grade, yet not in parents’ education level and household income. Age-related declines in MVPA, increases in ST and lower sleep duration in children have been demonstrated by previous evidence [2,50]. The relationship may be from the increased time that is required for academia as one progresses through school [2,51]. This might displace PA opportunities and reduce sleep duration [51]. Another explanation might be that older Chinese children have access to a greater number and type of electronic digital devices (e.g., smart phone, iPad and laptops), which may result in increased sedentary screen activities [52].

In this study, we also found a lower compliance with movement guidelines in girls, which is in line with previous studies of children in the mainland and Hong Kong [2,10]. Interestingly, we did not find significant differences in parental and family socioeconomics with respect to the guideline adherence, where mixed results were also indicated in previous studies [2,53]. Overall, the above findings suggest that future intervention design and policy-making should take age, grade and gender differences into consideration. For example, physical active break programs could be implemented to improve the PA and reduce the prolonged sedentary time in a school setting [54]. More strategies for motivating the girls to engage in daily PA are needed. In addition, more studies on examining the impacts of other demographic factors are needed.

Regarding the contribution of individual movement behavior on weight status and body composition, our study hypotheses were partially supported. Consistent with previous studies [12,16,55], engaging in more total PA and less ST was associated with lower levels of PBF and lower risks of overweight/obesity in children, respectively. Interestingly, a positive association between ST and FFM was found in our study, which is contrary to our hypothesis and previous evidence. This might be attributed to the inclusion of the weight status as a covariate. In the crude model, a negative association between ST and FFM was identified, whereas the association direction was changed when controlling for the weight status in the multivariate model. A potential explanation is that in our study, children who were overweight or obese showed more ST and these overweight/obese children had higher levels of FFM compared to the children with normal weight status.

It is worth noting that sleep duration was negatively associated with FFM and SMM in the crude model, whereas the association did not remain after including demographics, weight status and other movement behaviors, similar to previous studies [56,57]. However, another national survey, of Korean children, revealed that decreased sleep duration was significantly associated with increased risks in the highest quartile of PBF and the lowest quartile of skeletal muscle index in boys, and there was no significant association between sleep duration and obesity parameters, except waist circumference in girls [58]. The above findings imply the need for more examinations on the relationship between sleep and weight-related outcomes in children, especially taking the multidimensional components of sleep (e.g., patterns, quality) into account [50,59]. However, this is likely a less imperative target than PA and ST, given the mixed findings.

Regarding the combined effects of movement behaviors, consistent with previous evidence [2,24], primary school children who met PA + ST and PA + sleep showed lower risks of overweight/obesity and lower levels of PBF. In addition, we found that if more of the guidelines were met, less risks of overweight/obesity and lower levels of PBF were reported. This finding is consistent with previous studies of children from Hong Kong and other regions/countries [10,14,19,24], supporting the hypotheses. Interestingly, we did not find any association between guideline adherence and FFM and SMM. Since there is scarce evidence to examine this association, we are not able to make comparisons. More empirical studies addressing this issue and exploring the potential mechanisms are needed.

Several limitations should be noted. First, we used self-reported measures for behavioral variables. Although these questionnaires have been well validated in previous studies, the recall bias and social expectation effects cannot be ruled out. To ensure a more accurate investigation, the use of device-based behavioral measures (e.g., accelerometers, pedometers) is suggested in future research. Furthermore, we used a cross-section design, which cannot identify the causal relationship. Longitudinal and experimental designs are warranted in the future. In addition, although we applied random stratified sampling, our findings could only reflect the sample characteristics of selected city and future studies with the inclusion of more regions and with a comparison of different cultural contexts are warranted. Finally, the impacts of more sociodemographic, psychological, physical, parental, social and environmental factors were not examined in this study, which should be identified in future studies [60]. Despite these limitations, our findings add empirical evidence for 24 h movement behaviors and their physical health benefits among Chinese primary school children, which is contributable to future health promotion research and practice in children and adolescents.

## 5. Conclusions

This study explored the demographic characteristics of meeting 24 h movement guidelines and the association of movement behaviors with weigh status and body composition in Chinese primary school children. Several salient demographic correlates of guideline adherence were identified. Our findings supported the association of 24 h movement behaviors with children’s weight status and PBF, yet not with FFM and SMM. These findings suggest that future interventions and policymaking on health promotion in children should take age, gender and grade into consideration. Promoting a healthy lifestyle (high PA, less ST and guideline-level sleep duration) may have a positive impact on weight status and PBF in Chinese primary school children. Further research on examining the association of guideline adherence with FFM and SMM and identifying more influential correlates of guideline adherence is warranted.

## Figures and Tables

**Table 1:** Characteristics of the study sample.

	Total (*n* = 978)	Adherence (*n* = 255)	Non-Adherence (*n* = 723)	*p*	*Cohen d*
**Age (years), mean (SD)**	9.11 (1.39)	8.96 (1.31)	9.16 (1.42)	0.016	0.14
**Gender, *n* (%)**					
Boy	520 (53.2)	153 (60.0)	367 (50.8)	0.011	0.21
Girl	458 (46.8)	102 (40.0)	356 (49.2)		
**Grade, *n* (%)**					
Grade-1	154 (15.7)	35 (13.7)	119 (16.4)	0.017	0.13
Grade-2	203 (20.8)	63 (24.7)	140 (19.3)		
Grade-3	241 (24.6)	77 (30.2)	164 (22.6)		
Grade-4	168 (17.2)	38 (14.9)	130 (17.9)		
Grade-5	150 (15.3)	29 (11.4)	124 (17.1)		
Grade-6	62 (6.3)	13 (5.1)	49 (6.7)		
**Weight status, *n* (%)**					
Not overweight/obese	691 (70.7)	195 (76.5)	496 (68.6)	0.018	0.22
Overweight/obese	287 (29.3)	60 (23.5)	227 (31.4)		
**Body composition, mean (SD)**					
PBF (%)	21.01 (9.54)	19.42 (8.43)	21.56 (9.84)	0.002	0.23
FFM (kg)	26.85 (6.57)	25.74 (5.26)	27.24 (6.94)	<0.001	0.23
SMM (kg)	13.77 (3.86)	13.15 (3.14)	13.99 (4.06)	<0.001	0.22
**Father education level, *n* (%)**					
Below college	705 (72. 1)	186 (72.9)	519 (71.8)	0.72	0.03
College or above	273 (27.9)	69 (27.1)	204 (28.2)		
**Mother education level, *n* (%)**					
Below college	668 (68.3)	174 (68.2)	494 (68.3)	0.98	0.002
College or above	310 (31.7)	81 (31.8)	229 (31.7)		
**Yearly household income ^1^, *n* (%)**					
Low	348 (35.6)	88 (34.5)	260 (36.0)	0.22	0.09
Medium	348 (35.6)	83 (32.5)	265 (36.7)		
High	282 (28.8)	84 (32.9)	198 (27.4)		

PBF = percentage of body fat; FFM = fat-free mass; SMM = skeletal muscle mass; ^1^ Yearly household income, low: RMB < 84,000; medium: RMB 84,000–132,000; high: RMB > 132,000.

**Table 2:** Associations of individual movement behavior with weight status among the study sample (*n* = 978).

	Weight Status ^1^	
	Crude Model ^a^ *OR* (95%CI)	Multivariate Model ^b^ *OR* (95%CI)
**Age**	1.10 (0.84, 1.44)	0.70 (0.54, 0.92) **
**Gender**		
Girl	Reference	Reference
Boy	1.81 (1.37, 2.40) ***	2.00 (1.49, 2.70) ***
**Grade (reference: Grade-1)**		
Grade-1	Reference	Reference
Grade-2	1.71 (1.01, 2.87) *	2.43 (1.33, 4.42) **
Grade-3	2.25 (1.37, 3.69) ***	4.53 (2.28, 9.02) ***
Grade-4	2.48 (1.47, 4.19) ***	6.84 (2.92, 16.02) ***
Grade-5	2.28 (1.34, 3.90) **	10.45 (3.22, 33.91) ***
Grade-6	2.77 (1.43, 5.38) ***	14.17 (3.90, 51.48) ***
**Father education level**		
Blow college	Reference	Reference
College or above	1.37 (1.02, 1.85) *	1.51 (1.02, 2.25) *
**Mother education level**		
Blow college	Reference	Reference
College or above	1.03 (0.77, 1.38)	0.74 (0.50, 1.09)
**Yearly Household income ^2^**		
Low	Reference	Reference
Medium	0.85 (0.60, 1.20)	1.18 (0.83, 1.68)
High	1.02 (0.74, 1.41)	1.12 (0.74, 1.68)
**Individual movement behavior**		
Light PA (MET-h/day)	1.01 (0.99, 1.02)	N/A
MVPA (MET-h/day)	0.98 (0.97, 0.99) *	N/A
Total PA (MET-h/day)	1.00 (0.99, 1.00)	0.99 (0.99, 1.00) ^+^
ST (hs/day)	1.10 (0.93, 1.28)	1.22 (1.04, 1.42) *
Sleep (hs/day)	0.92 (0.77, 1.09)	1.04 (0.85, 1.28)

PA = physical activity; ST = screen time; ^1^ weight status: non-overweight/obese = 0, overweight/obesity = 1; ^2^ yearly household income, low: RMB < 84,000; medium: RMB 84,000–132,000; high: RMB > 132,000; ^a^ crude model: bivariable association between individual correlate variables and outcome variable; ^b^ multivariate model: adjusted for all demographic covariates in the multivariate model; N/A: not applicable; ^+^ *p* < 0.10; * *p* < 0.05; ** *p* < 0.01; *** *p* < 0.001.

**Table 3:** Associations of individual movement behavior with body composition among the study sample (*n* = 978).

	PBF (%)		FFM (kg)		SMM (kg)	
	Crude Model ^a^ *B* (95%CI)	Multivariate Model ^b^ *B* (95%CI)	Crude Model ^a^ *B* (95%CI)	Multivariate Model ^b^ *B* (95%CI)	Crude Model ^a^ *B* (95%CI)	Multivariate Model ^b^ *B* (95%CI)
**Age**	0.97 (0.54, 1.39) ***	0.28 (−0.38, 0.95)	3.34 (3.13, 3.55) ***	2.02 (1.59, 2.44) ***	1.96 (1.84, 2.08) ***	1.22 (0.97, 1.47) ***
**Gender**						
Girl	Reference	Reference	Reference	Reference	Reference	Reference
Boy	0.72 (−0.47, 1.92)	−0.93 (−1.69, −0.18) *	1.26 (0.44, 2.08) **	0.49 (0.01, 0.97) *	0.84 (0.36, 1.32) ***	0.40 (0.12, 0.69) **
**Grade**						
Grade-1	Reference	Reference	Reference	Reference	Reference	Reference
Grade-2	2.72 (0.78, 4.65) **	1.11 (−0.31, 2.54)	3.25 (2.28, 4.22) ***	0.64 (−0.27, 1.55)	1.92 (1.35, 2.49) ***	0.35 (−0.19, 0.88)
Grade-3	5.77 (3.90, 7.64) ***	3.07 (1.38, 4.76) ***	4.99 (4.05, 5.92) ***	1.07 (−0.01, 2.14)	2.94 (2.39, 3.49) ***	0.58 (−0.05, 1.21) ^+^
Grade-4	7.29 (5.27, 9.31) ***	4.15 (2.03, 6.27) ***	7.13 (6.12, 8.15) ***	1.98 (0.63, 3.33) **	4.22 (3.62, 4.81) ***	1.09 (0.30, 1.89) **
Grade-5	4.62 (2.56, 6.69) ***	1.93 (−1.05, 4.91)	13.36 (12.33, 14.40) ***	5.06 (3.17, 6.96) ***	7.79 (7.18, 8.40) ***	2.82 (1.71, 3.94) ***
Grade-6	7.59 (4.87, 10.31) ***	3.70 (.43, 6.97) *	15.54 (14.17, 16.90) ***	6.75 (4.66, 8.84) ***	9.21 (8.40, 10.01) ***	3.91 (2.68, 5.13) ***
**Father education level**						
Below college	Reference	Reference	Reference	Reference	Reference	Reference
College or above	2.01 (0.69, 3.34) **	0.42 (−0.62, 1.45)	0.65 (−0.27, 1.57)	−0.34 (−1.00, 0.32)	0.40 (−0.14, 0.94)	−0.17 (−0.56, 0.22)
**Mother education level**						
Below college	Reference	Reference	Reference	Reference	Reference	Reference
College or above	0.72 (−0.56, 2.01)	−0.03 (−1.02, 0.96)	0.07 (−0.82, 0.95)	0.22 (−0.41, 0.85)	0.06 (−0.46, 0.58)	0.14 (−0.24, 0.51)
**Yearly Household income ^1^**						
Low	Reference	Reference	Reference	Reference	Reference	Reference
Medium	−0.53 (−1.94, 0.88)	−0.12 (−1.02, 0.79)	−1.05 (−2.00, −0.10) *	0.36 (−0.22, 0.94)	−0.69 (−1.25, −0.14) *	0.14 (−0.20, 0.48)
High	−2.09 (−3.59, −0.60) **	−0.54 (−1.58, 0.50)	−3.94 (−4.94, −2.93) ***	0.45 (−0.22, 1.11)	−2.33 (−2.92, −0.74) ***	0.22 (−0.17, 0.61)
**Weight status**						
Non-overweight/obese	Reference	Reference	Reference	Reference	Reference	Reference
Overweight or obesity	16.02 (15.18, 16.86) ***	15.65 (14.81, 16.48) ***	6.23 (5.42, 7.05) ***	5.48 (4.95, 6.01) ***	3.65 (3.17, 4.13) ***	3.19 (2.88, 3.51) ***
**Individual movement behavior**						
Light PA (MET-hr/day)	0.01 (−0.02, −0.05)	N/A	0.05 (0.02, 0.07) ***	N/A	0.03 (0.02, 0.04) ***	N/A
MVPA (MET-hr/day)	−0.05 (−0.08, −0.03) ***	N/A	0.05 (0.04, 0.07) ***	N/A	0.03 (0.02, 0.04) ***	N/A
Total PA (MET−hr/day)	−0.03 (−0.05, −0.01) **	−0.03 (−0.04, −0.01) ***	0.05 (0.04, 0.07) ***	<0.01 (−0.01, 0.01)	0.03 (0.02, 0.04) ***	<0.01 (−0.01, 0.01)
ST (hr/day)	0.26 (−0.35, 0.87)	0.34 (−0.06, 0.74) ^+^	−0.47 (−0.89, −0.05) *	0.27 (0.01, 0.52) *	−0.31 (−0.55, −0.06) *	0.13 (−0.03, 0.28)
Sleep (hr/day)	−0.55 (−1.40, 0.30)	0.11 (−0.44, 0.65)	−1.82 (−2.40, −1.25) ***	−0.20 (−0.55, 0.15)	−1.02 (−1.36, −0.69) ***	−0.07 (−0.28, 0.14)

PBF = percent body fat; FFM = fat free mass; SMM = skeletal muscle mass; PA = physical activity; ST = screen time; ^1^ yearly household income, low: RMB < 84,000; medium: RMB 84,000–132,000; high: RMB > 132,000; ^a^ crude model: bivariable association between individual correlate variables and outcome variable; ^b^ multivariate model: adjusted for all demographic covariates and weight status in the multivariate model; N/A: not applicable; ^+^ *p* < 0.10; * *p* < 0.05; ** *p* < 0.01; *** *p* < 0.001.

**Table 4:** Associations of meeting 24 h movement guidelines with weight status and body composition among the study sample.

	Weight Status ^1^	PBF (%)	FFM (kg)	SMM (kg)
	*OR* (95%CI)	*B* (95%CI)	*B* (95%CI)	*B* (95%CI)
**Meeting (vs. not meeting) individual guideline**				
At least PA	0.66 (0.50, 0.88) **	−1.75 (−2.52, −0.98) ***	0.15 (−0.34, 0.65)	0.06 (−0.23, 0.35)
At least ST	0.72 (0.53, 0.97) *	−0.59 (−1.38, 0.21)	-0.39 (−0.89, 0.11)	−0.16 (−0.45, 0.14)
At least Sleep	1.07 (0.78, 1.48)	0.01 (−0.83, 0.84)	−0.53 (−1.16, 0.10)	−0.27 (−0.58, 0.04) ^+^
**Meeting (vs. not meeting) specific combination**				
PA + ST	0.67 (0.49, 0.90) **	−1.33 (−2.11, −0.54) ***	−0.21 (−0.71, 0.29)	−0.07 (−0.36, 0.23)
PA + Sleep	0.67 (0.50, 0.91) *	−1.14 (−1.93, −0.36) **	−0.26 (−0.76, 0.24)	−0.12 (−0.41, 0.17)
ST + Sleep	0.81 (0.61, 1.09)	−0.34 (−1.10, 0.42)	−0.48 (−0.96, 0.00) ^+^	−0.25(−0.53, 0.03) ^+^
All three	0.63 (0.44, 0.88) **	−0.79 (−1.65, 0.07) ^+^	−0.55 (−1.09, 0.00) ^+^	−0.30(−0.62, 0.02) ^+^
**Number of guidelines adhered to**				
Meet non/one	Reference	Reference	Reference	Reference
Meet two	0.90 (0.65, 1.25)	−1.50 (−2.37, −0.63) **	0.17 (−0.39, 0.72)	0.19 (−0.14, 0.52)
Meet all	0.59 (0.40, 0.87) **	−1.63 (−2.62, −0.65) **	−0.46 (−1.08, 0.17)	−0.19 (−0.56, 0.18)
Trend analysis	0.78 (0.65, 0.94) *	−0.85 (−1.34, −0.36) ***	−0.21 (−0.52, 0.11)	−0.08 (−0.26, 0.10)

All models were adjusted for age of children, gender, grade, parental education level and household income and/or weight status; PBF = percent body fat; FFM = fat free mass; SMM = skeletal muscle mass; PA = physical activity; ST = screen time; ^1^ weight status: non-overweight/obese = 0, overweight/obesity = 1; ^+^ *p* < 0.10; * *p* < 0.05; ** *p* < 0.01; *** *p* < 0.001.

## Data Availability

Requests for data and materials should be directed to the corresponding author or first author.
